# The Influence of Workplace Incivility on Employees’ Emotional Exhaustion in Recreational Sport/Fitness Clubs: A Cross-Level Analysis of the Links between Psychological Capital and Perceived Service Climate

**DOI:** 10.3390/healthcare7040159

**Published:** 2019-12-06

**Authors:** Chia-Ming Chang, Li-Wei Liu, Hsiu-Chin Huang, Huey-Hong Hsieh

**Affiliations:** 1 Department of Physical Education, Health & Recreation, National Chiayi University, Chiayi 62103, Taiwan; gr5166@yahoo.com.tw; 2 Department of Leisure Service Management, Chaoyang University of Technology, Taichung 41349, Taiwan; ijofsrm@gmail.com; 3 Physical Education and Arts School, Chengyi University College, Jimei University, Xiamen 361021, China; op5166@yahoo.com.tw; 4 Department of Leisure Management, Taiwan Shoufu University, Tainan 72153, Taiwan

**Keywords:** sport centers, positive psychology, hierarchical linear modeling, diary-method

## Abstract

The aim of this study was to explore the influence of workplace incivility on the emotional exhaustion of recreational sport/fitness club providers through a cross-level analysis. A total of 200 recreational sport/fitness club providers from Taiwan were selected for the repeated collection of measures and a 10-day diary method was used. The effect of workplace incivility on recreational sport/fitness club employees’ emotional exhaustion on a daily basis was analyzed at the intra-personal level, and the relationship between psychological capital and perceived service climate was studied at the inter-personal level. Five hypotheses were developed and tested using hierarchical linear modeling. The results found that employees’ emotional exhaustion and burnout highly correlated with workplace incivility and service climate. Based on the results, recommendations for employees and sport/fitness centers are proposed. Furthermore, research limitations and future directions are discussed.

## 1. Introduction

The market share of recreational sport/fitness clubs is consistently expanding and evolving. Over the years, private clubs, such as World Gym, BEING Sport, Curves, and Fitness Factory have become established in many different cities in Taiwan and continue to grow [1]. According to Lin and Teng [2], employees in the service-oriented industry are asked to conceal their emotions, put on a smile, and provide the best services for customers regardless of their internal feelings at all times. However, it is very likely that they may suffer emotional burnout under such circumstances. Emotional exhaustion often arises when employees are faced with difficult customers. It should be noted that the emotional exhaustion of service-providing employees can drive customers away, and results in a reduction of customers and sales [3]. Emotional burnout can have tremendously negative impacts on the job satisfaction and performance of employees, and eventually cause them to quit [4,5,6]. Therefore, this study aimed to explore the relationships among emotional exhaustion, job performance, emotion burnout and related factors, in recreational fitness and health club service staff.

The diary method, as Hershcovis and Reich [7] pointed out, collects self-repeated reports, because the same variables are observed over a longer period of time. Unlike cross-sectional data, repeated measures can capture the participants’ dynamic perspectives, attitudes, and feelings. Halbesleben and Wheeler [8] used a five-day diary method to study interpersonal workplace aggression from an integrated perspective. Through examining and critiquing current methods, measurement, and proposing different approaches, they concluded that the diary method can explore workplace aggression in a more dynamic and contextualizing way.

Employees at health and fitness centers are the key to service quality and customer satisfaction. However, the workplace incivility they face on a daily basis may be sabotaging their performance, and the negative effect of emotional exhaustion is largely overlooked. Therefore, the present study used the diary method to collect individual, intrapersonal perceptions of workplace incivility and emotional burnout, and analyzed the relationship between incivility and emotional burnout. In addition, the study also collected interpersonal data, such as psychological capital (PsyCap) and perceived service climate and performed a cross-sectional analysis to examine the sport/fitness clubs’ cross-level impacts on employee burnout.

## 2. Hypothesis Development

### 2.1. Work Incivility and Emotional Burnout

In an organizational context, affective event theory (AET) explains employees’ everyday mood and emotions [9]. Johonson and Indvic [10] pointed out that workplace incivility creates a hostile environment. Any simple incivility within the environment eventually turns out to be violence, hurting employees physically and mentally. Furthermore, Vickers [11] discovered that workplace incivility discourages teamwork, and the loss of team spirit is then accompanied by a subsequent sense of loneliness. For instance, when service providers deal with unfriendly demanding customers, they are usually stressed and hurt [12]. Incivility inside or outside the organization typically results in negative attitudes, which later affect employees’ job performance and job satisfaction [9]. If negative thoughts accumulate fast enough, employees are more like to feel emotionally exhausted and quit their job. Based on literature evidence, the following hypothesis was proposed:

**Hypothesis 1** **(H1).** *Workplace incivility has positive effects on emotional burnout.*

### 2.2. PsyCap and Emotional Burnout

PsyCap is characterized by four qualities: efficacy, optimism, hope and resilience [13,14]. As Locke and Latham [15] reported, individuals with higher self-efficacy were more likely to set and achieve high goals, and to attempt harder tasks. Similarly, Yu, Chen, and Hung [16] stated that employees with the same quality tended to perform well through difficult times. They analyzed situations, maintained composure, and outperformed themselves. In addition, Snyder [17] proposed that hope creates positive moods and attitudes which lead individuals to the right pathways (strategies) to reach goals. Scheier and Carver [18] indicated that optimistic people expected good things to happen. Based on the broaden-and-build theory, positive moods change individuals’ thinking and behavior, making them more creative and innovative. Positive moods enhance individuals’ physical, mental, social and psychological well-being, all of which increase employees’ competence [19]. Literature evidence concluded that employees with higher self-efficacy feel more capable of achieving goals, stay positive when facing challenges, select the right pathways to goals, and are thus less likely to feel emotional burnout.

**Hypothesis 2** **(H2).** *PsyCap has negative effects on emotional burnout.*

### 2.3. Work Incivility, PsyCap and Emotional Burnout

Workplace incivility happens all the time. Interrupting phone calls, expressing a lack of respect, and unfair treatment are typical examples of workplace incivility. It produces negative emotions, resulting in employees’ no longer wanting to contribute to the organization [20]. As Pugh [21] indicated, service providers were more likely to feel emotional exhaustion when they were asked to restrain their inner feelings and not show their authentic selves [22]. According to Tosten and Toprak [23], individuals high in PsyCap are generally optimistic, manage their emotions and handle life pressure well, and hope for the best. Furthermore, Afzal, Atta, and Malik [24] discovered that those with high levels of PsyCap were determined to achieve goals while facing challenges, and maintained high hopes while embracing struggles.

**Hypothesis 3** **(H3).** *PsyCap has a moderating effect on the relationship between workplace incivility and emotional exhaustion.*

### 2.4. Service Climate and PsyCap

The literature evidence suggested that PsyCap leads to positive moods, fosters personal well-being, increases organizational commitment, and improves job performance [13]. Schneider, White, and Paul [25] stated that the extent to which employees are rewarded for delivering quality service determines the strength of their organization’s service climate. In addition, as reported by Walumbwa, Peterson, Avolio, and Hartnell [26], an organization’s service climate has some level of effect on its employees’ PsyCap, which leads to high job performance. In other words, employees who perceive a high service climate have higher PsyCap. Liu [16] discovered that employees’ perceptions of the service climate have significant positive effects on their PsyCap. Therefore, the study proposed the following:

**Hypothesis 4** **(H4).** *Service climate has a significant positive effect on PsyCap.*

### 2.5. Service Climate and Emotional Burnout

The job demands–resources model proposes that an organization’s service climate requires employees to engage with job resources. Their engagement not only influences their mental health, but also increases their job performance [27]. Several studies have shown that emotional demands on service providers are typically high. Thus, a positive organizational service climate—amid workplace incivility—is a prominent interpersonal resource, and it buffers the impact of job demands on stress-reactions [28]. Importantly, perceived service climates help service providers at health and fitness clubs cope with workplace incivility, promote their perceived organizational support, foster improved job performance, and reduce stress and burnout.

**Hypothesis 5** **(H5).** *Perceived service climate has significant negative effects on emotional burnout.*

**Figure 1:**
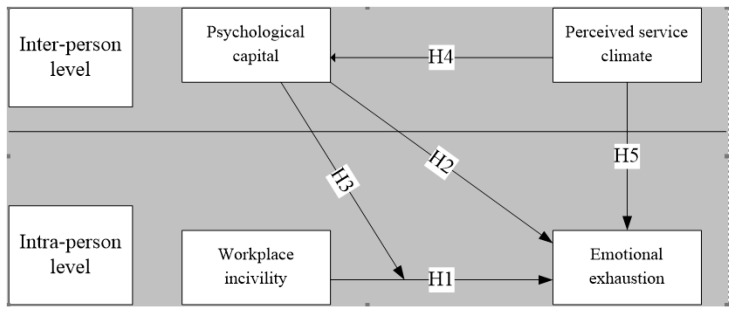
Research hypothesis model.

Figure 1 presents the research hypotheses framework.

## 3. Methods

### 3.1. Participants

The subjects of the research were members of the service staff at health and fitness centers located in different cities, including Taipei City, New Taipei City, Chiayi City, Chiayi County, Tainan City, and Kaohsiung City in Taiwan. A total of 2000 employees were selected to participate in the research. Data were collected using the 10-day diary method. In order to increase the response rate, a mobile text message was set to send to each individual participant at 17:00 every afternoon. All the subjects were informed about the data collection procedure when they were invited to participate in the research. Data were collected under a clearly informed consent agreement. The research was reviewed and approved by the Institutional Review Board. A total of 1790 valid responses were retrieved, with a valid return rate of 89.5%.

### 3.2. Research Instruments

Research participants were invited to complete their 10-day diary within a month. Workplace incivility and emotional burnout were assessed on a daily basis; therefore, data were retrieved for 10 consecutive days. On the other hand, PsyCap and service climate—which were individual level variables—were collected on the first day of participation and examined accordingly. The scales used in the research and demographic variables are presented as follows.

#### 3.2.1. Demographic Variables

Demographic variables were designed to meet the specific research needs. Demographic variables included gender (male = “1”, female = “2”), age, education level (middle school = “1”, high school = “2”, college = “3”, masters or above = “4”), marital status (not married = “1”, married = “2”), perceived health status (ranging from 1 = “bad” to 5 = “excellent”), and length of service (in years).

#### 3.2.2. Workplace Incivility Checklist

The workplace incivility checklist used in the present study was modified from Liu and Dai [29]. The checklist had 14 items, which consisted of five dimensions: exclusionary behavior, gossiping, hostility, privacy invasion, and abuse of power. Participants were asked to respond yes or no to formulated questions based on research dimensions. These questions included: Have you been in a situation where your supervisor/co-workers (a) doubted your judgment in a matter over which you have responsibility? (b) Addressed you privately in unprofessional terms? (c) Made demeaning, rude, or derogatory remarks about you? (d) Took away your office supplies without your permission and did not return them? (e) Treated you unfairly? Each “yes” answer counted as 1 point. The total score ranged from 0 to 14.

#### 3.2.3. Emotional Exhaustion Scale

The Emotional Exhaustion Scale was modified from the measurement of experienced burnout originally introduced by Maslach and Jackson [30]. Statements were rewritten to best suit the research needs. The four statements were: (a) I feel burn out from my work; (b) dealing with customers all day is really a strain for me; (c) I feel used up at the end of workday; (d) I feel emotionally drained from work today. All items were measured using a 5-point Likert scale, ranging from 1 being “strongly disagree”, to 5 being “strongly agree”. The confirmatory factor analysis (CFA) showed the model was a good fit (χ^2^ / df = 3.33, GFI = 0.98, CFI = 0.98, NNFI = 0.95, RMSEA= 0.07). Cronbach’s α was 0.900, indicating that the reliability of the scale was acceptable.

#### 3.2.4. Psychological Capital Scale

Psychological capital scale was adopted from Luthans, Youssef, and Avolio [14] and Yu et al. [16]. The scale comprised 16 questions, including four dimensions of self-efficacy, hope, optimism, and resilience. Sample statements based on four dimensions, respectively, were: (a) I feel confident that I can work with colleagues in a professional manner; (b) I am optimistic about what will happen to me in the future as it pertains to work; (c) I work as the goals set by the belief that where there is a will, there is a way; (d) I usually manage difficulties one way or another at work. All items were measured using a 5-point Likert scale, ranging from 1 being “strongly disagree”, to 5 being “strongly agree”. The confirmatory factor analysis (CFA) showed the model was a good fit (χ^2^ / df = 3.17, GFI = 0.93, CFI = 0.96, NNFI = 0.94, RMSEA= 0.07). Cronbach’s α was 0.907, indicating that the reliability of the scale was acceptable.

#### 3.2.5. Perceived Service Climate Scale

The perceived service climate scale was modified from Schneider et al. [25], which had four dimensions. However, a unidimensional global service climate was found in much literature [25,31,32]. Therefore, the scale had 7 questions, covering only one dimension. A sample statement was “Employees in our organization have knowledge of the job and the skills to deliver superior quality work and service”. All items were measured using a 5-point Likert scale, ranging from 1 being “strongly disagree”, to 5 being “strongly agree”. The confirmatory factor analysis (CFA) showed that the model was a good fit (χ^2^/df = 5.16, GFI = 0.90, CFI = 0.96, NNFI = 0.93, RMSEA= 0.09). Cronbach’s α was 0.927, indicating that the reliability of the scale was acceptable.

#### 3.2.6. Control Variables

The control variables which were considered to affect employees’ psychology and behavior were gender, age, education level, health status and length of service. Control variables were selected based on relevant research [29,33].

### 3.3. Data Analysis

Statistical analysis was performed using SPSS 21.0 for Windows (IBM SPSS software, CA, USA) [34] and HLM 7.0 (Scientific Software International, Inc., IL, USA) [35] Given the significance level of 0.05, hypothesis testing was conducted using descriptive statistics, confirmatory factor analysis, and hierarchical liner modeling.

## 4. Results

### 4.1. Data Aggregation and Variable Descriptive Statistics

Table 1 presents the mean (M), standard deviation (SD), and correlations of the aggregates within and between subject variables. As for workplace incivility (scores ranging from 0 (low) to ~14 (high)), most participants perceived low workplace incivility, which was good to know that most customers in gyms or fitness centers were nice customers. As for emotional exhaustion, most participants reported low emotional exhaustion (scores ranging from 1 (low) to 5 (high)). Among those participants (*n* = 1790), most of them were female (M = 1.68), married (M = 1.77), with a college degree (M = 3.04), at an age of around 30 years old (M = 30.42), and their average length of service was 5.29 years. As for perceived health status, most of them felt their health status was good (M=3.46, scores ranging from 1 = “bad” to 5 = “excellent”). As for psychological capital and perceived service climate, most participants had high psychological capital (M = 3.46), and perceived high service climate (M = 3.88).

### 4.2. Hypothesis Testing

Five research hypotheses were formulated and tested using hierarchical linear modeling. Results were presented as follows.

#### 4.2.1. Null Model Analysis

The research intended to analyze the effects of within-subject variables and between-subject variables on the emotional exhaustion of frontline service employees at health and fitness centers. The cross-level analysis was primarily verified using null model analysis. Bliese [36] suggested to use the intra-class correlation coefficients (ICCs) as indicators to test whether the data are appropriate for HLM analysis. Two types of ICCs can explain the aggregation levels of the data structure: ICC (1) represents the percentage of variance between groups and ICC (2) represents the reliability of the group mean scores. The ICC (1) value must be equal to or greater than 0.07, while ICC (2) values must be equal to or greater than 0.50 [37]. A Chi-square test in null model, presented in Table 2, indicated that between-subject variance in employees’ emotional exhaustion was significant (χ^2^ = 1487.98, df = 178, *p* < 0.001), and that ICC (1) was 0.424, indicating that the individual factor accounted for 42.4% of variance of employees’ emotional burnout. According to James [38], an ICC (1) greater than 0.12 indicates acceptable reliability; therefore, hierarchical liner modeling was used for data analysis. Moreover, Glick [39] suggested that ICC (2) is an index of the reliability of group. Measure was consistent with ICC (2) being greater than 0.60. The ICC (2) of workplace incivility was 0.853 and employee emotional burnout was 0.880, indicating high reliability. The level-1 and level-2 models for emotional exhaustion and workplace incivility, respectively, are expressed as follows:

Emotional Exhaustion

• Level-1 Model
_✓_ Emotional Exhaustion_ m_j_ = ψ_0j_ + em_j_
• Level-2 Model
_✓_ ψ_0j_ = γ_00_ + u_0j_
• Workplace incivility
• Level-1 Model
_✓_ Workplace incivility_ mj = ψ_0j_ + em_j_
• Level-2 Model
_✓_ ψ_0j_ = γ_00_ + u_0j_

#### 4.2.2. HLM Analysis

In Table 2, M1 reported that out of six control variables (gender, age, marital status, educational level, work tenure, and perceived health status), perceived health status was the only variable that had a significant effect on the emotional burnout of service providers at health and fitness centers (γ_06_ = −0.216). This indicated that perceived health status was negatively associated with emotional burnout.

Workplace incivility had significant positive effects on emotional exhaustion (*ψ_1_* = 0.230), indicating that H1 was supported. PsyCap, however, had significant negative effects on emotional burnout (γ_07_ = −0.318); thus, H2 was supported. Furthermore, PsyCap was found to have no mediating effects on the relationship between incivility and burnout. H3 was rejected. Lastly, H5 was supported because perceived service climate had significant negative effects on burnout (γ_08_ = −0.118).

• Level-1 Model
_✓_ Emotional Exhaustion_mj = ψ_0j_ + ψ_1j_* (Workplace incivility _mj_) + e_mj_
• Level-2 Model
_✓_ ψ_0j_ = γ_00_ + γ_01_*(Gender_M_j_) + γ_02_*(Age_M_j_) + γ_03_*(Marrige_M_j_) + γ_04_*(Eduation_M_j_) + γ_05_*(length of service_M_j_) + γ_06_*(Health_M_j_) + γ_07_* (PsyCap l_M_j_) + γ_08_*(Perceived service climate_M_j_) + u_0j_
_✓_ ψ_1j_ = γ_10_+ γ_11_*(PsyCap _M_j_)

**Figure 2:**
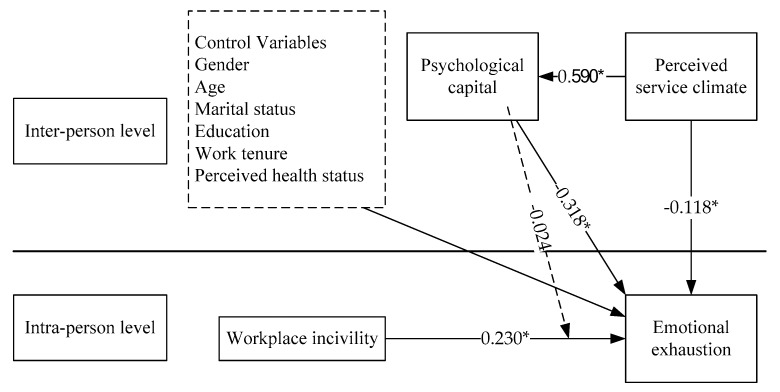
Results of research hypothesis test (*: *p* < 0.05.)

The results of regression reported the significant positive effect of PsyCap on the perceived service climate (β = 0.590, *p* < 0.05). Higher levels of PsyCap were associated with higher levels of perceived service climate. Results of hypothesis testing are presented in Table 2 and Figure 2.

### 4.3. Discussion

#### 4.3.1. Effects of Control Variables on Emotional Exhaustion

The research results reported that, out of five control variables (gender, age, marital status, education level, length of service, and perceived health status), perceived health status was highly associated with emotional exhaustion. Service providers who considered themselves as having a high perceived health status were less likely to experience emotional burnout. Similarly, health care professionals with higher physical health perception were found to have lower fatigue and stress [40]. Individuals with high perceived health status in general were less likely to experience job burnout [41]. Additionally, Chen, Davis, Daraiseh, Pan, and Davis [42] reported that nurses who exercised every week had less fatigue and better recovery outcome.

#### 4.3.2. Workplace Incivility and Emotional Exhaustion

The 10-day diary method was utilized to collect repeated measures from service staff at health and fitness clubs. With five control variables controlled, research findings indicated that workplace incivility might cause employees’ emotional exhaustion. The findings were consistent with affective events theory (AET), that claims that workplace incivility might influence employees’ everyday emotion and behaviors [9]. Cortina [43] also found that workplace incivility was negatively associated with job performance, work atmosphere and team morale. Incivility—which is defined by Anderson and Pearson [44] as low-intensity deviant behavior with ambiguous intent to harm the target, in violation of workplace norms for mutual respect—takes many forms. When compared to physical harassment, incivility is typically thought to be low intensity; therefore, it draws little attention and becomes prevalent [43,45]. Pearson and Porath [46] discovered that prevailing incivility in a workplace tended to hurt individual employees and teams as a whole, disengaged employees, and sabotaged team cohesiveness [47]. In line with our research, these findings suggest that workplace incivility should not be treated lightly.

Three common causes of workplace incivility are abusive supervision, employee mistreatment or discrimination, and disrespectful customers [48,49]. The first two causes come within the organization, while the third cause comes from outside. While it is widely believed that employees’ commitment to service and professionalism makes a great first impression, which creates an image in the mind of customers, the reality is far from the truth. Because professional development training is overlooked and underappreciated as an employee recruitment tool, organizations find it difficult to hire full-time employees who are willing to contribute [50]. Incivility has negative consequences in the workplace and remains a prevailing issue in service industry.

#### 4.3.3. Relationships among PsyCap, Workplace Incivility and Emotional Exhaustion

Throughout the cross-level analysis, the result indicated that PsyCap had no moderating effect on the relation between workplace incivility and emotional burnout. PsyCap, on the other hand, was found to have significant negative effects on emotional exhaustion, and so did perceived service climate. PsyCap, according to Luthans, Youssef, and Avolio [14], is composed of four positive capacities, namely self-efficacy, optimism, hope and resilience. The first capacity is self-efficacy, a sense of self-confidence in one’s own ability and the sense that one has the capacity to put forward the effort to achieve a goal. Optimism, the second element, is the sense that one can succeed both now and in the future, and is based on the concept that positive events are internal. The third element is hope, and it is the sense of individual agency, or control, to work toward one’s goals. The final attribute is resilience, characterized as one’s positive ability to cope with the adversity or stress often found in conflicts or failures, the idea being that one can bounce back to attain success when faced with deep adversity or challenge.

#### 4.3.4. Service Climate and Emotional Burnout

The results showed that service climate had a negative impact on emotional burnout. An encouraging service climate plays a vital role in service quality [25]. The findings were consistent with the job demands resources model that claims service climate as being a resource engagement that strengthens employee relationships and buffers the negative impact of job demands on burnout [28]. Moreover, a good service climate makes individual employees fully aware of the purpose of their job and the meaning behind it and enhances their work engagement [51]. According to Crawford, LePine, and Rich [52], a positive service climate makes employees face challenges energetically to become completely involved in their jobs.

#### 4.3.5. Service Climate and PsyCap

Results showed that service climate was found to be significantly positively related to PsyCap, which is consistent with Liu’s assertion [16] that positive service climates elevated employees’ PsyCap levels.

## 5. Conclusions

### 5.1. Summary

According to our findings, first, we found that among five control variables (gender, age, marital status, education level, length of service, and perceived health status), perceived health status was positively correlated with emotional exhaustion. Second, we found that workplace incivility might cause employees’ emotional exhaustion. Third, throughout the cross-level analysis, the result indicated that PsyCap had no moderating effect on the relationship between workplace incivility and emotional burnout. PsyCap, on the other hand, was found to have significant negative effects on emotional exhaustion, and so did perceived service climate. Fourth, service climate had negative impacts on emotional burnout and, finally, service climate was found significantly positively related to PsyCap.

### 5.2. Suggestion

According to the findings, suggestions have been made for managerial implications. As for frontline employees at sport and fitness centers, it is suggested to use the facilities at the workplace which can improve their health and decrease emotional exhaustion. As for workplace incivility, the human resources department may establish appropriate procedures to assist employees in coping with difficult people to decrease employees’ emotional exhaustion. As for PsyCap, people were not born with PsyCap. In fact, PsyCap can be developed through learning. Therefore, for any individual or organization, the recognition and development of PsyCap could help develop better self-awareness [53]. Individuals can invest in learning to strengthen their cognitive abilities, self-management skills and personality traits to reduce emotional exhaustion [54]. As for the service climate, organizations can establish a unique positive service climate through training programs, performance appraisals and systems for rewarding good performance to reduce employees’ emotional burnout. As for the relationship between service climate and PsyCap, it is encouraged that the management at health and fitness centers create a positive service climate to increase their employees’ PsyCap.

### 5.3. Limitations

Upon the first day of the participants’ recruitment, all respondents promised to complete the daily investigation. However, there may have been some tired-out employees forgetting to complete the daily investigation and leaving their responses to the very next day, or even just simply using the next day’s perception. In any case, we strived to encourage the participants to complete the daily investigation to avoid this confusion.

## Figures and Tables

**Table 1:** Descriptive statistics of the variables and correlation coefficients within subjects and between subjects.

Variable	Mean	SD	Correlation							
			1	2	3	4	5	6	7	8
Within Subjects										
1.Workplace Incivility	0.37	0.96								
2.Emotional Exhaustion	2.00	0.80								
Between Subjects										
1.Gender	1.68	0.47	1							
2.Age	30.42	7.11	–0.26 *	1						
3.Marital Status	1.77	0.42	0.22 *	–0.54	1					
4.Education Level	3.04	0.39	0.05	0.10	0.00	1				
5.Length of Service	5.29	5.01	–0.16	0.71 *	–0.35 *	0.20 *	1			
6.Perceived Health Status	3.46	0.69	–0.25	0.04	–0.01	0.03	0.04	1		
7.Psychological Capital	3.61	0.49	–0.22	0.24 *	–0.29 *	0.03	0.23 *	0.28 *	1	
8.Perceived Service Climate	3.88	0.68	–0.19	–0.03	–0.09	–0.09	0.02	0.12	0.59 *	1

Note: within subjects: *n* = 1790; between subjects: *n* = 179; M: mean; SD: standard deviation; *: *p* < 0.05.

**Table 2:** Control variables, workplace incivility, psychological capital and perceived service climate predict emotional exhaustion by HLM.

	Emotional Exhaustion	
	M0	M1
Intercept	2.000 *	1.913 *
Within Subjects		
Workplace Incivility (ψ_1_)		0.230 *
Between Subjects		
Gender (γ_01_)		0.014
Age (γ_02_)		–0.004
Marital Status (γ_03_)		0.014
Education Level (γ_04_)		0.101
Work Tenure(γ_05_)		0.007
Perceived Health Status (γ_06_)		–0.216 *
PsyCap (γ_07_)		–0.318 *
Perceived Service Climate (γ_08_)		–0.118 *
Cross-level		
PsyCap * Workplace Incivility (γ_11_)		0.024
σ^2^	0.368	0.341
τ_00_	0.271	0.150
τ_01_	0.368	0.340
Deviance	3670.59	3497.94

Note: within subjects: *n* = 1790; between subjects: *n* = 179; *: *p* < 0.05; the meanings of the remaining Greek symbols can be found in the HLM models.
